# Acne and PCOS are less frequent in women with Mayer-Rokitansky-Küster-Hauser syndrome despite a high rate of hyperandrogenemia: a cross-sectional study

**DOI:** 10.1186/1477-7827-12-23

**Published:** 2014-03-18

**Authors:** Katharina Rall, Gabriele Conzelmann, Norbert Schäffeler, Melanie Henes, Diethelm Wallwiener, Matthias Möhrle, Sara Y Brucker

**Affiliations:** 1 Department of Obstetrics and Gynecology, Tübingen University Hospital, Calwerstrasse 7, 72076 Tübingen, Germany; 2 Department of Dermatology, Tübingen University Hospital, Liebermeisterstrasse 25, 72076 Tübingen, Germany; 3 Division of Psychosomatic Medicine and Psychotherapy, Department of Internal Medicine VI, Tübingen University Hospital, Osianderstrasse 5, 72076 Tübingen, Germany; 4 Praxisklinik Tübingen Haut und Venen, Europaplatz 2, 72072 Tübingen, Germany

**Keywords:** MRKH, Acne, Quality of life, Dermatology life quality index (DLQI), Hyperandrogenemia, WNT4, PCOS

## Abstract

**Background:**

Acne is a very common skin condition during adolescence and adulthood. Patients with uterovaginal agenesis (Mayer-Rokitansky-Küster-Hauser syndrome, MRKH) treated at the Tübingen University Center for Rare Female Genital Malformations, however, clinically appeared to be less frequently affected by acne. The etiology of MRKH syndrome remains unknown. The only known MRKH-associated mutations are located within the *WNT4* gene and lead to an atypical form of MRKH syndrome associated with clinical and biochemical hyperandrogenism. Our study aimed to assess the frequency, severity, and self-evaluation of acne in MRKH patients and to correlate the clinical findings with hormone analyses.

**Methods:**

As part of a cross-sectional longterm follow-up study after laparoscopic assisted creation of a neovagina a questionnaire was sent to 149 MRKH patients aged 16–44 years comprising 26 items concerning prevalence and self-evaluation of acne, and the effects of acne on quality of life. The questionnaire was derived from one used in a former epidemiological study of acne in 4,000 women. Blood for hormone analyses was collected routinely during the clinical visit.

**Results:**

Fully completed, evaluable questionnaires were returned by 69/149 (46%) women. Of these respondents, 42 (60.1%) showed hyperandrogenemia without other clinical signs of virilization but only 17 (24.6%) reported acne (8 (11.6%) had physiological acne and 9 (13.0%) clinical acne) and only 10 (14.5%) reported receiving medical treatment for their acne. Effects of acne on quality of life were minor. Only 4 patients (5.8%) with PCOS were identified, among them one with physiological acne, the other three within the acne-free group.

**Conclusions:**

Although hyperandrogenemia is common, acne is significantly less frequent in women with MRKH than reported in the literature for non-MRKH women, and is seldom treated medically. Patients in this study appeared resistant to acne to some extent, possibly due to the sebaceous glands in the acne regions being less sensitive to androgens compared to the normal population. A *WNT4* mutation is unlikely to be the main cause of MRKH syndrome in our hyperandrogenemic patients.

## Background

Mayer-Rokitansky-Küster-Hauser (MRKH) syndrome is characterized by congenital uterovaginal agenesis [1,2], resulting from cessation of müllerian duct development around the fifth week of gestation [3]. The incidence of MRKH syndrome is estimated at 1 in 4,500 female births [4]. Despite ongoing research, the etiology of this syndrome has begun to be elucidated for some women, but is still unknown for the majority of patients.

Since women with the syndrome have a normal 46,XX karyotype and functioning ovaries, they show normal physical development and manifestation of secondary sexual characteristics [1,2]. Compared to controls, MRKH patients are generally similar in hormone levels and can be grouped into cyclic phases [4-6]. In the majority of cases there are no clinical or biochemical signs of hyperandrogenism [2].

Due to familial clustering, the possibility of genetic causes has been the subject of continued study. MRKH syndrome does not present with clearly identifiable genetic causes except in a Wnt4-associated atypical form with clinical and biological signs of hyperandrogenism [7]. In mice, the *Wnt4* gene regulates female reproductive tract development and antagonizes testosterone production [8]. The *WNT4* mutation has been reported to be associated with failure of müllerian duct formation and virilization, including acne, in at least four 46,XX women [9,10]. In fact, it appears that, at least in a subgroup of MRKH patients, the absence of the vagina and uterus and excess androgen levels are the pathognomonic signs of *WNT4* defects, causing a clinical entity distinct from the typical MRKH syndrome [11,12].

At our Center for Rare Female Genital Malformations, a joint division of the Department of Gynecology and the Treatment and Research Center for Rare Diseases at the University of Tübingen, we provide in close collaboration with the Division of Psychosomatic Medicine at our university hospital medical treatment, psychological counseling, and postsurgical follow-up examinations to more than 40 MRKH patients per year [13].

Acne vulgaris is the most common skin disease worldwide. Various recent epidemiological studies undertaken in recent years have shown that acne is very common in women from mid-to- late adolescence, as well as during adulthood [14-16]. Primarily a disease of adolescence, acne is triggered by the initiation of adrenal and gonadal androgen production and usually subsides after the end of growth [16-18]. At our institution, MRKH patients appeared to have less acne than expected during routine follow-up, despite being in the predisposed age group.

Acting as an endocrine organ, the sebaceous gland responds to changes in androgens and other hormones [19,20]. Although levels of circulating androgens are mostly normal, sebaceous glands in acne regions are more sensitive to androgens than in other parts of the body [21].

There is a genetic predisposition to acne. However, the specific mechanisms of inheritance remain unclear [22,23].

There are two main assessment aspects, the first being the objective activity of the disease (visible signs) and the second being its impact on quality of life. Although more than 25 different methods have been developed to measure acne, it remains difficult to do so objectively because there is no general agreement as to the most suitable method [19].

Polycystic ovary syndrome (PCOS) is a reproductive and metabolic disorder affecting 5-10% of women in their reproductive age [24]. A refined definition of PCOS was agreed, namely the presence of two of the following three features: oligo- and/or anovulation, which can be hardly determined in MRKH syndrome, hyperandrogenism, and polycystic ovaries [25]. An increased prevalence of PCOS and Müllerian anomalies has already been described [26].

The aim of the present study was to determine the frequency, severity and self-evaluation of acne in women with MRKH and to investigate the potential relationship between clinical and biochemical signs of androgen excess including the prevalence of PCOS. A potential correlation between MRKH and reduced acne prevalence may contribute to a better understanding of the etiology of MRKH syndrome, especially with respect to the role of the Wnt signaling cascade.

## Methods

### Study design and participants

This was part of a cross-sectional study in which a questionnaire was sent out by postal mail in April 2010 to 149 women with MRKH syndrome treated at the Centre for Rare Genital Malformations, a division of the Department of Obstetrics and Gynecology at Tübingen University Hospital, Germany, as a longterm follow-up after laparoscopic assisted creation of a neovagina. The primary aim of this part of the study was to assess the presence of clinical acne, physiological acne, or no acne. We received prior approval from the Ethics Committee of Tübingen University Hospital.

Patients included in the study had proven MRKH syndrome, a normal female 46,XX karyotype, normal pubertal development, and normal secondary sexual characteristics. There were no patients with clitoral hypertrophy or hirsutism. Hormonal work-up (normal ranges) included total testosterone (<0.35–1.4 nmol/L below 21 years of age and 0.4–2.1 nmol/L over 21 years), luteinizing hormone (0.5–76.3 IU/L), follicle-stimulating hormone (1.5–33.4 IU/L), androstenedione (1.17–12 nmol/L), dehydroepiandrosterone sulfate (DHEAS; 0.8–11.5 umol/L), progesterone (16–2000 nmol/L), estradiol (70–1300 pmol/L) and 17-hydroxyprogesterone (17-OHP; 0.61–4.97 nmol/L). Hyperandrogenemia was defined as elevated testosterone levels with or without elevated androstenedione, 17-OHP, and/or DHEAS levels.

The incidence of polycystic ovary syndrome (PCOS) was determined by evaluation of the routinely performed ultrasound, pelvic magnetic resonance imaging (MRI) as well as by analyzing the surgical reports.

### Questionnaire

The questionnaire comprised 26 items and was based on a French questionnaire used in a 2001 epidemiological survey of acne in 3,394 adult women aged 25–40 years [16]. The original French questionnaire was validated and contained 40 questions relating to skin type, lifestyle, factors influencing or preventing acne, current skin care practices, and the effect of acne lesions on quality of life (Dermatology Life Quality Index (DLQI) [27]. The original French questionnaire was translated into German by a native German dermatologist and validated through back translation by a native French and German speaker (see Additional file 1). To assess quality of life, the questionnaire was complemented with the German version of the Finlay DLQI questionnaire, used with kind permission of Dr. M. Basra, Department of Dermatology, Cardiff University [28].

Essentially, participants were asked whether they had had acne between the ages of 12 and 20 years and also whether they had had acne after age 20 years. They were also asked to count the number of currently active pustules, papulonodules, blackheads and closed comedones in defined areas of the face.

Acne severity was categorized “clinical acne”, “physiological acne” or “no acne” according to a clinical index modified from [16]. The main modification consisted in limiting the information requested to the date of the questionnaire rather than covering the prior 3-month period. Thus, the following definitions were used:

• Clinical acne: ≥ 5 pustules or papulonodules on the face (except nose) with or without blackheads at the date of the questionnaire, *or* > 2 pustules or papulonodules on the face (except nose) with blackheads at the date of the questionnaire

• Physiological acne: 1 or 2 pustules or papulonodules on the face (except nose) with or without blackheads at the date of the questionnaire

• No acne: no pustules or papulonodules, and no blackheads at the date of the questionnaire.

### Statistical analysis

Statistical analysis was performed using SPSS software version 17.0. The Kruskal-Wallis test was used to compare MRKH patients with and without acne for differences in risk factors. Spearman’s rho was used to correlate acne severity and effects on quality of life. The chi-square test was used to detect significant differences in acne prevalence between women with and without MRKH syndrome from the literature. Significance was given if the p-value was below 0.05.

## Results

### Study cohort characteristics

The demographic and clinical characteristics of the study cohort are summarized in Table 1.

**Table 1:** Characteristics of MRKH women in a self-report questionnaire survey on acne

	**Clinical acne**	**Physiological acne**	**No acne**	**All respondents**
***Invited participants, n***	**—**	**—**	**—**	149
***Respondents, n***	**—**	**—**	**—**	71
***Respondents returning evaluable questionnaires, n***	9	8	52	69
*Demographic and clinical characteristics*				
***Mean age ± SD, years (n = 69)***	23.6 ± 6.6	23.1 ± 5,3	23.3 ± 6.6	23.3 ± 6.4
***Median age [range], years (n = 69)***	21.0 [18-39]	21.5 [17-33]	21.0 [16-44]	21.0 [16-44]
***Inflamed lesions (at date of questionnaire), mean no.***	7.8	1.9	n.a.	n.a.
***Inflamed lesions (at date of questionnaire), median no. [range]***	6 [3-17]	2 [1-3]	n.a.	n.a.
***Medical treatment for acne, n***	1	0	9	10
*Androgen status*				
Hyperandrogenemia, n (%*)	6 (14.3%)	6 (14.3%)	30 (71.4%)	42 (100%)
No hyperandrogenemia, n (%*)	2 (14.3%)	0 (0%)	12 (85.7%)	14 (100%)
Incomplete hormonal work-up, n (%*)	1 (7.7%)	2 (15.4%)	10 (76.9%)	13 (100%)

— no data.

n.a. not applicable.

*percent of row total.

By 31 July 2010, the study cut-off date, 71 (48%) of the 149 invited MRKH women had responded. Of the other 78 women, 3 had declined to participate in the survey, and 19 had not received their questionnaires as these were returned as undeliverable.

Of the 71 questionnaires returned, 69 were evaluable whilst 2 were incomplete. Respondents were 16–44 (mean 23.3, median 21.0) years of age. 31 patients had associated malformations, mainly of the kidneys and skeletal system.

### Acne prevalence and severity

Of the 69 evaluable respondents, 52 (75.4%) reported being free of acne at the date of questionnaire completion. The remaining 17/69 (24.6%) women reported having acne, which was categorized as clinical acne in 9/69 (13.0%) and as physiological acne in 8/69 (11.6%) women. The acne severity score, as determined by counting specific skin lesions, tended to be slightly higher in MRKH patients compared to other acne patients from the literature. The ratio between clinical and physiological acne was nearly 1:1. Thus about 50% of our respondents affected by acne had physiological acne. There was no correlation between hyperandrogenemia or acne (Table 1) and a more severe MRKH phenotype with associated malformations.

MRKH women with clinical acne on the cheeks mainly had blackheads. Pustules were present on the forehead but completely missing from the chin and nose. Papulonodules occurred predominantly on the chin, while closed comedones occurred predominantly on the nose.

In the women with clinical acne, the mean number of inflamed lesions at the date of the questionnaire was 7.8 (median [range], 6 [3-17]) whilst it was 1.9 (2 [1-3]) in those with physiological acne (Table 1).

All MRKH women with clinically overt clinical acne and physiological acne reported manipulating (squeezing) their lesions.

Out of the 7 participants aged ≥21 years with clinical acne, 2 (29%) had not had acne during their teens. Combining clinical and physiological acne, 3 out of 13 women (23%) had not had acne during their teens.

Risk and stress factors, e.g. smoking, quality of sleep and use of make-up were also investigated. A Kruskal-Wallis test showed that MRKH women with acne did not differ significantly from MRKH women without acne in respect of daily cigarette consumption, quality of sleep and use of make-up.

### Treatment

Only 10 (14%) out of all 69 evaluable respondents reported receiving medical treatment for their acne lesions (Table 1). One of the 9 (11.1%) respondents with clinical acne and none of the 8 (0%) respondents with physiological acne reported having received such treatment. Of the 52 women who reported being acne-free, 9 (17%) had received medical treatment for their acne, of whom 4 (44%) reported having had clinical acne between the age of 12 and 20.

The following concomitant medications were reported: hormonal contraceptives, 3/69 (4%); other systemic hormones, 3/69 (4%); topical estrogen cream in the genital area for other reasons, 2/69 (3%); and antibiotics, 5/69 (7%).

### Hyperandrogenemia

The results of the hormonal work-up are shown by acne category in Table 1. As many as 42/69 (60.1%) showed hyperandrogenemia without other clinical signs of virilization. Serum testosterone levels ranged between 1.5 and 4.0 nmol/L. Of the acne-free women, 30/52 (57.7%) had biochemical signs of hyperandrogenism, 12/52 (23.1%) had normal results, and 10/52 (19.2%) only had an incomplete hormonal work-up. Hyperandogenemia was present in 6 (75%, 2 unknown) patients with physiological acne and 6 patients (66.7%, 1 unknown) with clinical acne. Normal androgen levels were observed in 2 (22.2%) of the 9 patients with clinical acne and none of the patients with physiological acne.

### Prevalence of PCOS

Evaluating ultrasounds and pelvic MRIs and retrospective analysis of the surgical reports identified only 4 patients (5.8%) with PCOS. Among them was one with physiological acne, the other three were within the acne-free group, in 6 patients the morphology of the ovaries was unknown, while in 59 patients (85.5%) the criteria for polycystic ovaries were not met.

### Quality of life

**Figure 1:**
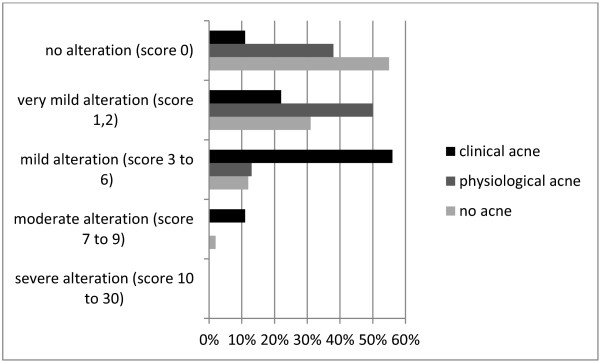
**Effect of clinically overt acne lesions on quality of life.** Analysis by category of self-reported acne severity using the Dermatology Life Quality Index (DLQI) (according to [16]).

The DLQI was used to investigate the relationship between quality of life and severity of acne. Results ranged from “no alteration” to “moderate reduction”. There was a significant difference between the groups with “clinical acne”, “physiological acne”, and “no acne” (*F* (2.65) = 6.591, *p* = 0.002). Post-hoc tests showed that the “clinical acne” group (mean score ± standard deviation (SD) 3.76 ± 2.50, n = 9) differed significantly from both the “no acne” group (1.25 ± 1.91, n = 52; *p* = 0.003) and the “physiological acne” group (0.88 ± 0.99, n = 8; *p* = 0.011). There was no significant difference between the “no acne” and “physiological acne” groups (*p* = 0.861, see Figure 1).

Despite the high percentage of hyperandrogenemia the chi-square test showed that at 24.6% (17 of 69 respondents), the prevalence of acne in the adolescent and adult women with MRKH participating in our study was significantly lower than in comparable cohorts of women and girls without MRKH, for example in Iranian adolescents (acne prevalence 92%, *p* <0.0001) [14], adolescents in Hong Kong (acne prevalence 81.5%, *p* <0.0001) [15] and French adolescents (acne prevalence 41%, *p* =0.04) [16].

## Discussion

This is the first study to report an unexpectedly low rate of clinical signs of androgen excess in a sample of 69 women with MRKH syndrome, as many as 60.8% of which had biochemical hyperandrogenemia. Androgen excess is one of the most common endocrinopathies, affecting approximately 7% of reproductive-aged women, the majority with a diagnosis of polycystic ovary syndrome (PCOS) and both clinical and biochemical evidence of hyperandrogenism. Clinically, hyperandrogenism manifests primarily as acne, androgenetic hair loss, and hirsutism. However, an element of observer bias in the evaluation of signs of hirsutism and clinical hyperandrogenism has been discussed in this context [29-32].

Interestingly, acne as a clinical sign of androgen excess was reported only by 12 (28.6%) of the 42 hyperandrogenemic women amongst the 69 respondents in our study. No other signs of hyperandrogenism were observed. The incidence of PCOS in our patients was not higher than described in the general population [24]. While nearly all cases of *WNT4* mutations reported in the literature to date had clinical and biochemical signs of androgen excess as pathognomonic signs [8-12], these signs were not seen in our cohort. There was also no correlation between MRKH syndrome with associated malformations and hyperandrogenemia or acne. Recent data suggest that *WNT4* mutations cause specific regulation of androgen synthesis enzymes. Thus, according to one study, the absence of a *WNT4* mutation in four adolescent girls with MRKH syndrome and clinical and biochemical hyperandrogenism strongly suggests the potential involvement of other constituents of the Wnt4/β-catenin signaling pathway [10]. We therefore conclude that a *WNT4* mutation is unlikely to be the cause of MRKH syndrome in the majority of our hyperandrogenemic patients.

According to many epidemiological studies evaluating current skin status in adolescents, acne prevalence ranges between 80% more than 90% [14,15,33]. Two studies reported mean prevalence in adults as 31.9% and 54% [17,34]. Considering that our survey included MRKH women aged 16 to 44, acne prevalence would have been expected to be much higher than the 24.6% observed in this study. Despite the high percentage of hyperandrogenemia the chi-square test showed that at 24.6%, the prevalence of acne in the adolescent and adult women with MRKH participating in our study was significantly lower than in comparable cohorts of women and girls without MRKH [14-16].

Thus about 50% of our respondents affected by acne had physiological acne, whereas other studies have reported higher percentages of physiological acne [16,35]. On the other hand, compared to the French study by Poli et al. [16], we found that the severity score including counted skin lesions was an average 1.6 points and 1.3 points higher in our patients with clinical and physiological acne, respectively.

Medical treatment of acne was infrequent and non-specific in our sample of MRKH women. Only 4% of all women took oral contraceptives, which are associated with a significantly lower prevalence of acne. Oral contraceptives may modify the androgenic effect and thus influence both the prevalence and severity of acne [36,37]. Whereas 55% of adolescents aged 14–19 years [38] and one third of all fertile women in Germany use oral contraceptives, the percentage of MRKH patients taking oral contraceptives is very low, this being due to infertility. In view of this, one would have expected acne to be even more prevalent among the adolescents with MRKH as oral contraceptives are effective in treating acne and can be considered a preventive treatment. Paradoxically, the highest percentage of acne treatment in our study was found in the respondents without acne. One possible explanation for this finding could be that these women had had acne at some time and had been treated successfully.

Analysis of the questionnaire results did not reveal any correlation between risk and stress factors, e.g. nicotine use, quality of sleep, make-up use, and the prevalence or severity of acne. The effect of nicotine consumption on acne has been addressed in various studies. Some found a positive correlation between the daily number of cigarettes smoked and acne, while in others there was no correlation [39,40]. According to the Federal Statistical Office of Germany, the percentage of smokers among 15 to 45–year-old females was 15–30.4% in 2009 [41]. Although our sample of MRKH included 35% smokers, acne prevalence was far below average.

The menstrual period, presumed to be one of the most important aggravating factors in acne [42,43], is naturally missing in MRKH. However, women with MRKH syndrome have functional ovaries and therefore experience exactly the same cyclic hormone changes as women without the syndrome but have a low prevalence of acne. While the acne questionnaire used in our study did not specifically ask about cyclically recurring symptoms, this question was generally addressed at the routine follow-up visits after neovagina surgery.

With respect to the correlation between quality of life factors and acne severity, the DLQI indicated only a slight decrease in quality of life. No respondent reported severe changes in quality of life. Several studies have reported a similar degree of “emotional impairment” as reported by patients with chronic disabling asthma, epilepsy, diabetes, arthritis [44], or psoriasis [45]. In our study, quality of life was only mildly to moderately affected by acne, the highest observed score being 9 out of 30. This relatively low score may be considered plausible, however, in view of the great impact on quality of life associated with the absence of the uterus and vagina and the infertility this entails.

A limitation of the present study is the low rate of returned questionnaires. This was likely due in part to the fact that the MRKH syndrome is a rare condition, but may partly also be explained by the observation that women with MRKH tend to avoid constant confrontation with their condition once they have undergone surgical treatment.

As pointed out in the 2012 European evidence-based (S3) guidelines for the treatment of acne, there are inherent difficulties in objectively measuring acne. No international consensus has been reached on a uniform classification system for acne. However, this diversity and the frequent lack of validation limits the direct comparability of different trials [19,46,47]. Counting acne lesions – particularly currently inflamed lesions, as in our study – is a widespread and reliable method of classifying acne [48,49].

Further common grading systems, such as the Global Acne Grading System (GAGS) [50], the Leeds acne grading system [51], and the German guidelines [52] assess current skin status but do not consider a patient’s medical history and lifestyle. Therefore our acne definition also refers exclusively to current skin status. The study definitions for assessing the presence or absence of acne were confirmed by high concordance (72%) between self-assessment (“Do you have acne?”) and the acne index of current skin status. In contrast, there was only a 45% agreement between self-assessment and a definition based on the anamnestic criterion from the French study by Poli et al. [16] as to the number of pustules or papulonodules patients had had on their face during the last three months. The latter study, according to which the prevalence of acne in French women was 41%, cannot be directly compared with our study because of the acne definition used in that study. We used the same clinical criteria but focused on currently active lesions whereas the French study additionally covered the acne history of the preceding three months. However, their question as to the number of facial pustules or papulonodules occurring during the preceding three months may have been ambiguous since “during” can be interpreted cumulatively as meaning “in total”, or, in terms of prevalence, as “once or at the same time” within the past 3 months. Adding the same anamnestic aspects to the current acne definition used in our study revealed a large discrepancy between the current skin status and that of the preceding three months. With 36 out of 55 (65%) women in the acne group reporting no current acne lesions, this would have resulted in a cumulative overestimation of previous skin lesions.

## Conclusions

In conclusion, acne appears to be significantly less frequent in women with MRKH syndrome than in women without the syndrome and is seldom treated medically. To date the cause of MRKH is unknown. As only 28.6% of the 60.8% of MRKH women with hyperandrogenemia in our study had acne as a clinical sign of androgen excess, a mutation within the *WNT4* gene cannot be expected in the majority of our hyperandrogenemic patients. The association of MRKH and clinical signs of hyperandrogenism requires further investigation. Further research is required to elucidate the relationship between MRKH syndrome and the low frequency of acne despite the high frequency of hyperandrogenemia in MRKH women. The syndrome could be related to a genetic aberration, for example, a gene mutation that not only causes the MRKH phenotype but also partly prevents the skin from developing acne. This aberration could cause androgen resistance similar to that in complete androgen insensitivity syndrome.

## Abbreviations

17-OHP: 17-hydroxyprogesterone; DHEAS: Dehydroepiandrosterone sulfate; DLQI: Dermatology life quality index; GAGS: Global acne grading system; MRKH: Mayer-Rokitansky-Küster-Hauser syndrome; PCOS: Polycystic ovary syndrome; QoL: Quality of life; SD: Standard deviation.

## Competing interests

The authors declare that they have no competing interests.

## Authors’ contributions

KR, GC, MM and SYB conceived of the study. KR, NS, MH, DW, SYB, and MM participated in the design and coordination of the study. KR and GC recruited the patients, distributed the questionnaires, and compiled the raw data from the returned questionnaires. GC, NS, and KR performed the statistical analysis. KR, GC, and SYB drafted the manuscript. NS, MH, DW and MM revised the draft manuscript for important intellectual content. All authors read and approved the final manuscript.

## Supplementary Material

Additional file 1 German acne questionnaire. Click here for file
