# Supplementary material for: Acne and PCOS are less frequent in women with Mayer-Rokitansky-Küster-Hauser syndrome despite a high rate of hyperandrogenemia: a cross-sectional study
Source: Reprod Biol Endocrinol. 2014 Mar 18;12:23. doi: 10.1186/1477-7827-12-23 (PMC4003801; doi:10.1186/1477-7827-12-23)
Supplement: Additional file 1 — German acne questionnaire. [file 1477-7827-12-23-S1.doc]

**Additional file1- German acne questionnaire**

**Aknefragebogen**

Die folgenden Fragen beziehen sich auf den Zustand Ihrer Haut sowie mögliche beeinflussende Faktoren und eventuelle Belastungen, die dadurch entstehen können.

Bitte lesen Sie die einzelnen Fragen aufmerksam durch und beantworten Sie jede der Fragen, indem sie ein zutreffendes Kästchen ankreuzen oder Ihre Antwort aufschreiben. Bitte kreuzen Sie – sofern nicht ausdrücklich anders angegeben – nur jeweils **eine Antwortmöglichkeit** an.

| **1** | **Wie alt sind Sie?** |  |
| --- | --- | --- |
|  | *________* Jahre |  |
|  |  |  |
| **2** | **Wie reagieren Sie wenn Sie sich ohne Sonnenschutz der Sonne aussetzen?** |  |
|  | ❑ Ich bräune, ohne einen Sonnenbrand zu bekommen | 1 |
|  | ❑ Ich bräune, aber manchmal bekomme ich einem Sonnenbrand | 2 |
|  | ❑ Ich bräune kaum, bekomme meist einen Sonnenbrand | 3 |
|  | ❑ Ich bräune nicht (und bekomme immer einen Sonnenbrand) | 4 |
|  | ❑ Ich setze mich nie der Sonne aus | 5 |
|  |  |  |
| **3** | **Hatten Sie Akne im Alter zwischen 12 und 20 Jahren?** |  |
|  | ❑ Ja | 1 |
|  | ❑ Nein | 2 |
|  |  |  |
| **4** | **Hatten Sie Akne nach Abschluss des 20. Lebensjahres** |  |
|  | ❑ Ja | 1 |
|  | ❑ Nein | 2 |
|  |  |  |
| **5** | **Wie war die Qualität Ihres Schlafes innerhalb der letzten 3 Monate** |  |
|  | ❑ Sehr gut | 1 |
|  | ❑ Ziemlich gut | 2 |
|  | ❑ Ziemlich schlecht | 3 |
|  | ❑ Sehr schlecht | 4 |
|  |  |  |
| **6** | **Rauchen Sie?** |  |
|  | ❑ Mehr als 10 Zigaretten am Tag | 1 |
|  | ❑ Zwischen 5 und 10 Zigaretten am Tag | 2 |
|  | ❑ Weniger als 5 Zigaretten am Tag | 3 |
|  | ❑ Ich rauche nicht | 4 |
|  |  |  |
| **7** | **Welche Medikamente haben Sie in den letzten 3 Monaten eingenommen**  **(auch Hormonpräparate, „Pille“, Beruhigungsmittel angeben)** |  |
|  | Name des Medikaments eintragen |  |
|  | a: __________________________ |  |
|  | b: __________________________ |  |
|  | c: __________________________ |  |
|  | d: __________________________ |  |
|  |  |  |

| **8** | | **Schminken Sie sich das Gesicht (Puder, Tönung) (nicht nur Lider oder Lippen)?** | | | | | |  |
| --- | --- | --- | --- | --- | --- | --- | --- | --- |
|  | | ❑ Immer | | | | | | 1 |
|  | | ❑ gelegentlich | | | | | | 2 |
|  | | ❑ selten | | | | | | 3 |
|  | | ❑ nie | | | | | | 4 |
|  | |  | | | | | |  |
| **9** | | **Auf der folgenden Zeichnung wurden bestimmte Gesichtspartien mit einer Nummer gekennzeichnet. Beschreiben Sie den jetzigen Zustand Ihrer Haut an jeder dieser Stellen.** | | | | | |  |
|  | |  | | | | | |  |
|  | | 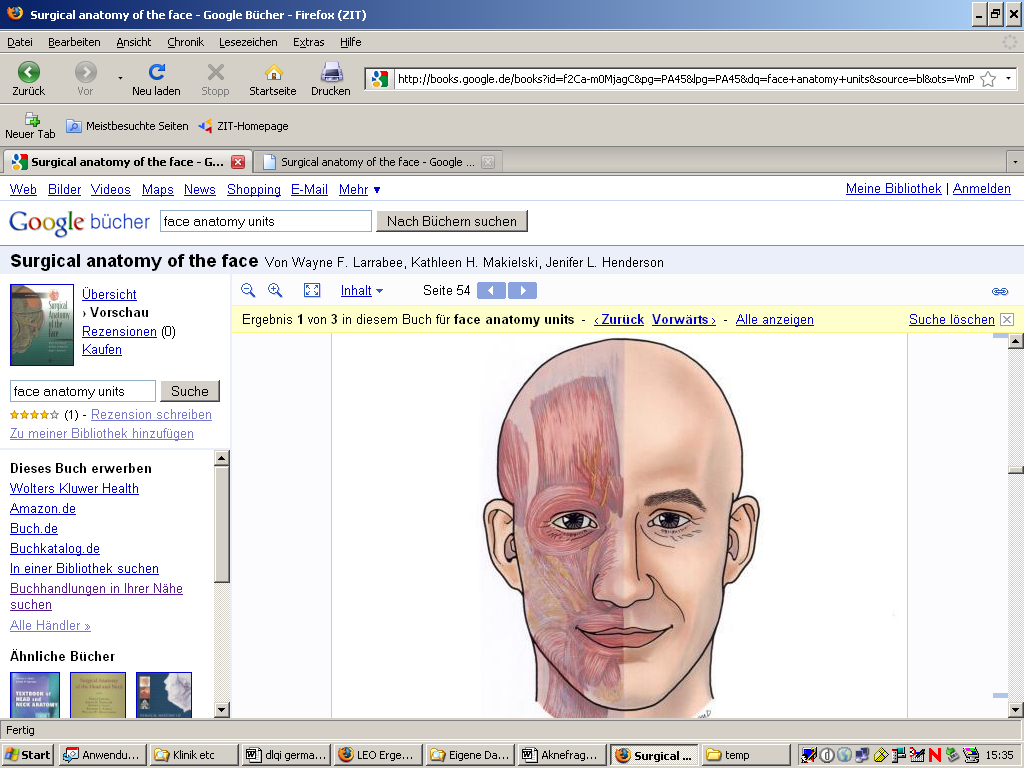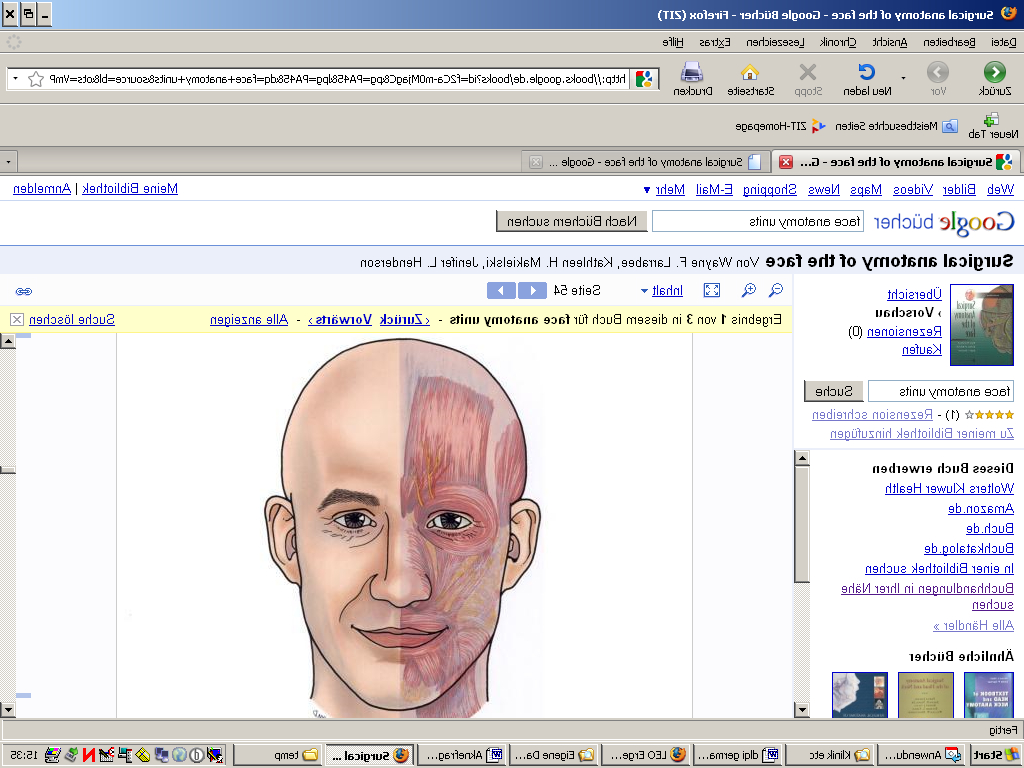 1. Stirn  5.Nase  2. obere Wange  2. obere Wange  4.Kinn  3. untere  Wange  3. untere  Wange | | | | | |  |
|  | | |  |  |  |  |  | |
|  | | | 1. Stirn | 2. obere Wangen | 3. untere Wangen | 4. Kinn | 5. Nase | |
| Offene Poren | | | ❑ Ja 1  ❑ Nein 2 | ❑ Ja 1  ❑ Nein 2 | ❑ Ja 1  ❑ Nein 2 | ❑ Ja 1  ❑ Nein 2 | ❑ Ja 1  ❑ Nein 2 | |
| Schwarze Mitesser | | | ❑ Ja 1  ❑ Nein 2 | ❑ Ja 1  ❑ Nein 2 | ❑ Ja 1  ❑ Nein 2 | ❑ Ja 1  ❑ Nein 2 | ❑ Ja 1  ❑ Nein 2 | |
| Pickel unter der Haut | | | ❑ Ja 1  ❑ Nein 2 | ❑ Ja 1  ❑ Nein 2 | ❑ Ja 1  ❑ Nein 2 | ❑ Ja 1  ❑ Nein 2 | ❑ Ja 1  ❑ Nein 2 | |
| Anzahl **schmerzhafter** Pickel **unter** der Haut | | |  |  |  |  |  | |
| Anzahl roter Pickel mit weißem „Kopf“ (weiße Mitesser) | | |  |  |  |  |  | |
|  |  | | | | | | |  |
| **10** | **Hatten Sie während der letzten 3 Monate an irgendeiner Stelle auf der obigen Skizze rote schmerzhafte Pickel unter der Haut oder rote Pickel mit weißem „Kopf“ (weiße Mitesser)?** | | | | | | |  |
|  | ❑ Nein, keine | | | | | | | 1 |
|  | ❑ Zwischen 1 und 4 Pickel | | | | | | | 2 |
|  | ❑ Zwischen 5 und 10 Pickel | | | | | | | 3 |
|  | ❑ Mehr als 10 Pickel | | | | | | | 4 |
|  |  | | | | | | |  |

| **11** | **Wie ist zur Zeit Ihre Haut außerhalb des Gesichts; am Hals, am Halsausschnitt, am Rücken?** | | | | | | | | |  |
| --- | --- | --- | --- | --- | --- | --- | --- | --- | --- | --- |
|  |  | | | | | | | | |  |
|  | | | Hals | | Halsausschnitt | | Rücken | | | |
| Offene Poren | | | ❑ Ja 1  ❑ Nein 2 | | ❑ Ja 1  ❑ Nein 2 | | ❑ Ja 1  ❑ Nein 2 | | | |
| Schwarze Mitesser | | | ❑ Ja 1  ❑ Nein 2 | | ❑ Ja 1  ❑ Nein 2 | | ❑ Ja 1  ❑ Nein 2 | | | |
| Pickel unter der Haut | | | ❑ Ja 1  ❑ Nein 2 | | ❑ Ja 1  ❑ Nein 2 | | ❑ Ja 1  ❑ Nein 2 | | | |
| Anzahl **schmerzhafter** Pickel **unter** der Haut | | | ❑ Ja 1  ❑ Nein 2 | | ❑ Ja 1  ❑ Nein 2 | | ❑ Ja 1  ❑ Nein 2 | | | |
| Anzahl roter Pickel mit weißem „Kopf“ (weiße Mitesser) | | | ❑ Ja 1  ❑ Nein 2 | | ❑ Ja 1  ❑ Nein 2 | | ❑ Ja 1  ❑ Nein 2 | | | |
|  |  | | | | | | | | |  |
| **12** | **Haben Sie im Gesicht, am Halsausschnitt oder am Rücken Narben nach Akne in Form von** | | | | | | | | |  |
|  | Dellen | ❑ Ja 1  ❑ Nein 2 | | | | | | | |  |
|  | Braunen Flecken | ❑ Ja 1  ❑ Nein 2 | | | | | | | |  |
|  |  | | | | | | | | |  |
| **13** | **Wenn Sie Pickel haben, kratzen Sie diese oder drücken Sie diese aus?** | | | | | | | | |  |
|  | ❑ Ja 1  ❑ Nein 2 | | | | | | | | |  |
|  |  |
|  |  | | | | | | | | |  |
| **14** | **Benutzen Sie zur Zeit ein Medikament gegen ihre Pickel** | | | | | | | | |  |
|  | ❑ Ja 1  ❑ Nein 2 (gehen Sie zu Frage 17 auf der nächsten Seite) | | | | | | | | |  |
|  |  |
|  |  | | | | | | | | |  |
| **15** | **Handelt es sich um** | | | | | | | | |  |
|  | ❑ Ausschließlich eine Lokaltherapie (Creme, Gel, Lotion…) | | | | | | | | | 1 |
|  | ❑ Ausschließlich eine systemische Behandlung mit Tabletten, Kapsel, Saft…. | | | | | | | | | 2 |
|  | ❑ Sowohl eine Lokaltherapie für die Haut **und** eine Behandlung mit Tabletten | | | | | | | | | 3 |
|  |  | | | | | | | | |  |
| **16** | **Welches Medikament benutzen Sie zur Zeit *und* wer hat es Ihnen verschrieben?** | | | | | | | | |  |
|  |  | | | | | | | | |  |
|  | Name des Medikaments | | | verschrieben durch | | | | | | |
|  | Creme, Gel, Lotion | | | Hautarzt | | Frauenarzt | | Hausarzt | | |
|  | __________________________ | | | ❑ 1 | | ❑ 2 | | ❑ 3 | | |
|  | __________________________ | | | ❑ 1 | | ❑ 2 | | ❑ 3 | | |
|  | __________________________ | | | ❑ 1 | | ❑ 2 | | ❑ 3 | | |
|  | __________________________ | | | ❑ 1 | | ❑ 2 | | ❑ 3 | | |
|  | Tabletten, Kapseln, Saft | | | Hautarzt | | Frauenarzt | | Hausarzt | | |
|  | __________________________ | | | ❑ 1 | | ❑ 2 | | ❑ 3 | | |
|  | __________________________ | | | ❑ 1 | | ❑ 2 | | ❑ 3 | | |
|  | __________________________ | | | ❑ 1 | | ❑ 2 | | ❑ 3 | | |
|  |  | | | | | | | |  | |

| **Ziel der folgenden Fragen ist es, herauszufinden, wie sehr Ihre Hauterkrankung Ihr Leben in den vergangenen 7 Tagen beeinflusst hat. Bitte kreuzen Sie zu jeder Frage ein Kästchen an.** | | |
| --- | --- | --- |
|  |  |  |
| **17** | Wie sehr hat Ihre Haut in den vergangenen 7 Tagen **gejuckt**, war **wund**, hat **geschmerzt** oder **gebrannt**? |  |
|  | ❑ sehr | 1 |
|  | ❑ ziemlich | 2 |
|  | ❑ ein bisschen | 3 |
|  | ❑ überhaupt nicht | 4 |
|  |  |  |
| **18** | Wie sehr hat Ihre Hauterkrankung Sie in den vergangenen 7 Tagen **verlegen** oder **befangen** gemacht? |  |
|  | ❑ sehr | 1 |
|  | ❑ ziemlich | 2 |
|  | ❑ ein bisschen | 3 |
|  | ❑ überhaupt nicht | 4 |
|  |  |  |
| **19** | Wie sehr hat Ihre Hauterkrankung Sie in den vergangenen 7 Tagen bei **Einkäufen** oder bei **Haus-** oder **Gartenarbeit** behindert? |  |
|  | ❑ sehr | 1 |
|  | ❑ ziemlich | 2 |
|  | ❑ ein bisschen | 3 |
|  | ❑ überhaupt nicht | 4 |
|  | ❑ Frage betrifft mich nicht | 0 |
|  |  |  |
| **20** | Wie sehr hat Ihre Hauterkrankung die Wahl der **Kleidung** beeinflusst, die Sie in den vergangenen 7 Tagen getragen haben? |  |
|  | ❑ sehr | 1 |
|  | ❑ ziemlich | 2 |
|  | ❑ ein bisschen | 3 |
|  | ❑ überhaupt nicht | 4 |
|  | ❑ Frage betrifft mich nicht | 0 |
|  |  |  |
| **21** | Wie sehr hat Ihre Hauterkrankung in den vergangenen 7 Tagen Ihre **Aktivitäten** **mit anderen Menschen** oder Ihre **Freizeitgestaltung** beeinflusst? |  |
|  | ❑ sehr | 1 |
|  | ❑ ziemlich | 2 |
|  | ❑ ein bisschen | 3 |
|  | ❑ überhaupt nicht | 4 |
|  | ❑ Frage betrifft mich nicht | 0 |
|  |  |  |
| **22** | Wie sehr hat Ihre Hauterkrankung es Ihnen in den vergangenen 7 Tagen erschwert, **sportlich** aktiv zu sein? |  |
|  | ❑ sehr | 1 |
|  | ❑ ziemlich | 2 |
|  | ❑ ein bisschen | 3 |
|  | ❑ überhaupt nicht | 4 |
|  | ❑ Frage betrifft mich nicht | 0 |
|  |  |  |

| **23** | Hat Ihre Hauterkrankung in den vergangenen 7 Tagen dazu geführt, daß Sie Ihrer **beruflichen** **Tätigkeit** nicht nachgehen oder nicht **studieren** konnten? |  |
| --- | --- | --- |
|  | ❑ ja | 1 |
|  | ❑ nein | 5 |
|  | ❑ Frage betrifft mich nicht | 0 |
|  |  |  |
|  | Falls "nein", wie sehr ist Ihre Hauterkrankung in den vergangenen 7 Tagen ein Problem bei Ihrer **beruflichen Tätigkeit** bzw. Ihrem **Studium** gewesen? |  |
|  | ❑ ziemlich | 2 |
|  | ❑ ein bisschen | 3 |
|  | ❑ überhaupt nicht | 4 |
|  |  |  |
| **24** | Wie sehr hat Ihre Hauterkrankung in den vergangenen 7 Tagen Probleme im Umgang mit Ihrem **Partner**, **Freunden** oder **Verwandten** verursacht? |  |
|  | ❑ sehr | 1 |
|  | ❑ ziemlich | 2 |
|  | ❑ ein bisschen | 3 |
|  | ❑ überhaupt nicht | 4 |
|  | ❑ Frage betrifft mich nicht | 0 |
|  |  |  |
| **25** | Wie sehr hat Ihre Hauterkrankung in den vergangenen 7 Tagen Ihr **Liebesleben** beeinträchtigt? |  |
|  | ❑ sehr | 1 |
|  | ❑ ziemlich | 2 |
|  | ❑ ein bisschen | 3 |
|  | ❑ überhaupt nicht | 4 |
|  | ❑ Frage betrifft mich nicht | 0 |
|  |  |  |
| **26** | Inwieweit war die **Behandlung** Ihrer Haut in den vergangenen 7 Tagen für Sie mit Problemen verbunden (z. B. weil die Behandlung Zeit in Anspruch nahm oder dadurch Ihr Haushalt unsauber wurde)? |  |
|  | ❑ sehr | 1 |
|  | ❑ ziemlich | 2 |
|  | ❑ ein bisschen | 3 |
|  | ❑ überhaupt nicht | 4 |
|  | ❑ Frage betrifft mich nicht | 0 |
|  |  |  |
|  |  |  |
|  | **Bitte prüfen Sie, ob Sie wirklich ALLE Fragen beantwortet haben. Vielen Dank!** |  |
|  |  |  |
|  | Frage 17-26  **©** A Y Finlay, G K Khan April 1992* (Text). **©** W J Loo November 2000* (Illustration). Vervielfältigung ohne Genehmigung der Autoren untersagt. |  |
